# Association between body mass index and survival outcomes for cancer patients treated with immune checkpoint inhibitors: a systematic review and meta-analysis

**DOI:** 10.1186/s12967-020-02404-x

**Published:** 2020-06-12

**Authors:** Yue An, Zhonghua Wu, Ningning Wang, Zhidong Yang, Yue Li, Boyang Xu, Mingjun Sun

**Affiliations:** 1 Department of Gastroenterology, The First Hospital of China Medical University, 155 Nanjing bei Road, Shenyang, Liaoning 110001 People’s Republic of China; 2 Department of Surgical Oncology and General Surgery, Key Laboratory of Precision Diagnosis and Treatment of Gastrointestinal Tumors, Ministry of Education, The First Affiliated Hospital of China Medical University, Shenyang, People’s Republic of China; 3 Department of Laboratory Medicine, The First Hospital of China Medical University, Shenyang, Liaoning 110001 People’s Republic of China

**Keywords:** Immune checkpoint inhibitors, Body mass index, Overall survival, Progression-free survival

## Abstract

**Background:**

Immune checkpoint inhibitors (ICIs) have been increasingly applied in the treatment of several kinds of malignancies. Some clinical demographic characteristics were reported to be associated with the ICIs efficacy. The purpose of our current meta-analysis was to clearly evaluated the relationship between BMI and ICIs efficacy for cancer patients receiving immunotherapy.

**Methods:**

A systematic search of Pubmed, EMBASE and conference proceedings was performed to investigate the influence of BMI on ICIs efficacy. Pooled analysis for overall survival (OS), progression-free survival (PFS) and immune-related adverse effects (IRAEs) were analyzed in current study.

**Results:**

A total of 13 eligible studies comprising 5279 cancer patients treated with ICIs were included in the analysis. The pooled analysis showed there is positive association between high BMI and improved OS and PFS among patients with ICIs treatment (OS: HR = 0.62, 95% CI 0.55–0.71, P < 0.0001; I^2^ = 26.3%, P = 0.202); PFS: HR = 0.71, 95% CI 0.61–0.83, P < 0.0001; I^2^ = 0%, P = 0.591). There is no significant difference between the incidence of all grade IRAEs between obese, overweight patients and normal patients (Overweight vs Normal: pooled RR = 1.28, 95% CI 0.76– 2.18, P = 0.356; Obese vs Normal: pooled RR = 1.36, 95% CI 0.85– 2.17, P = 0.207).

**Conclusion:**

An improved OS and PFS were observed in patients with high BMI after receiving ICIs treatment compared with patients of low BMI. No significant association between BMI and incidence of IRAEs was found in cancer patients after ICIs treatment.

## Background

Immune checkpoint inhibitors (ICIs) targeting programmed cell death 1 (PD-1), its ligand 1 (PD-L1) and cytotoxic T lymphocyte-associated antigen 4 (CTLA-4), including nivolumab, atezolizumab, and ipilimumab etc., have been increasingly applied in treating several kinds of cancers because of their evident efficacy [1, 2]. Although the use of ICIs has brought survival benefits to cancer patients, the efficacy of ICIs varies widely among cancer patients during clinical practice with some patients experiencing a poor prognosis because of ICIs resistance [3–5]. Under such circumstance, a considerable amount of studies have been carried out to identify predictive biomarkers which may help recognizing patients who could benefit from ICIs [6–8]. Currently, available predictive biomarkers for ICIs response, such as PD-L1 expression, tumor mutation burden, microsatellite instability and tumor infiltrating lymphocytes (TILs) are mainly confined to cancer itself or its associated TILs [6, 9]. However, the patients receiving ICIs immunotherapy are of high heterogeneity and tumor-based biomarkers are not fully validated. Recently, several patient-associated demographic characteristics are being investigated to estimate the efficacy of ICIs [10]. One such characteristic, body mass index (BMI) OR obesity, caught much attention.

Obesity, defined by high body mass index (BMI) (≥ 30 kg/m^2^) according to the WHO standard definition, is a well-established risk factors for many malignancies, tumor progression, tumor recurrence etc. [11, 12]. However, some recent retrospective studies showed that high BMI possessed an positive association with improved overall survival (OS) and progression-free survival (PFS) in cancer patients receiving immunotherapy [13, 14]. Moreover, some preclinical data also revealed that obesity increases amount of aged T cell with higher PD-1 expression and dysregulated immune function, which could make cancer cells more sensitive to ICIs therapy [15]. Although several researches have investigated the association between the BMI and ICIs efficacy, heterogeneous conclusions were revealed in these studies [16, 17]. Therefore, a high-grade meta-analysis was required to evaluate the relationship between patients BMI and ICIs efficacy.

The purpose of our current meta-analysis was to clearly evaluate the relationship between BMI and ICIs efficacy for cancer patients receiving immunotherapy.

## Methods

### Study search

A systematic literature search was performed to identify relevant studies which have evaluated the association between BMI and ICIs efficacy for cancer patients through the Pubmed and Embase (up to January 2020) through the following search strategy: “nivolumab”, “pembrolizumab”, “avelumab”, “atezolizumab”, “lambrolizumab”, “pidilizumab”, “durvalumab”, “ipilimumab”, “tremelimumab”, “immune checkpoint inhibitor”, “PD-1 inhibitor”, “PD-L1 inhibitors”, “CTLA-4 inhibitors”, “cancer”, “tumor”, “neoplasm”, “carcinoma”, “BMI”, “Body Mass Index”, “Quetelet Index”, “Quetelet’s Index”, “Quetelets Index”, “obesity”, “overweight”, “weight”, “Mass”. In addition, references of relevant articles were also carefully reviewed to identify potentially eligible studies. For an attempt to find more relevant unpublished data, the abstracts on American Society of Clinical Oncology (ASCO) annual meeting, the European Society for Medical Oncology (ESMO) congress and Chinese Society of Clinical Oncology (CSCO) annual meeting were further reviewed.

### Study selection and quality assessment

Two authors (Yue An and Zhonghua Wu) independently reviewed all the terms to identify eligible studies. The goal of our current meta-analysis was to assess the association between BMI and ICIs efficacy in cancer patients. For this purpose, we included eligible studies according to the following inclusion criteria: (1) eligible patients were diagnosed with a solid cancer and treated with ICIs alone or ICIs combined with systemic chemotherapy. However, cancer patients treated with ICIs accompanied with loco-regional therapy were not included; (2) BMI was reported in eligible studies and was categorized into groups according to cutoff values; (3) The survival outcome was reported and measures could be extracted from the texts. When duplicated data was reported in different studies, we will only include the most recent or highest quality study. The quality of the included studies was assessed according to the Newcastle–Ottawa scale (NOS) scale [18].

### Data extraction

Two authors (Yue An and Zhonghua Wu) independently reviewed and extracted data from the included studies. Any discrepancy was resolved through discussion with the third author. The following data was extracted from all the included studies: (1) the name of first author and year of publication; (2) sample size, cancer types and types of ICIs drugs. (3) BMI values and the cutoff value for stratifying high or low BMI patient population; (4) Hazard ratio (HR) associated OS, PFS and their corresponding 95% CI. The HR for multivariate cox analysis was the top priority for use when reported. Any discrepancy during the data extraction was resolved through discussion.

### Statistical analysis

The primary outcomes were OS and PFS, and the association between BMI and ICIs efficacy was measured by HR with the corresponding 95% CI. The pooled HR and corresponding 95% CI were then calculated to evaluate the association between BMI and ICIs efficacy in cancer patients. The HR < 1.0 suggests improved OS and PFS in patients with high BMI, and HR > 1.0 indicates reduced PFS and OS. The pooled relative risk (RR) was analyzed to assess the relationship between BMI and the incidence rate of immune-related adverse effects (IRAEs) in cancer patients receiving ICIs treatments.

Statistical heterogeneity across included studies was evaluated using the Cochrane Q test and I^2^ statistic, and I^2^ > 50% and/or P < 0.05 was considered statistically heterogenous [19]. A random-effects model was chosen for meta-analysis if there was significant heterogeneity between studies. Otherwise, a fixed-effects model was selected [20]. Subgroup analyses were performed to evaluate the influence of cancer type on the influence of BMI on ICIs efficacy. Publication bias was assessed using Begg’s and Egger’s tests [21, 22].

All statistical analyses were performed based on Stata software (Version 12.0, Stata Corporation, College Station, TX, USA). A statistical significance was recognized if the 95% CI of pooled HR did not cover 1.0, with a *P* value less than 0.05 (two-sided).

## Results

### Characteristics of eligible studies

**Fig. 1:**
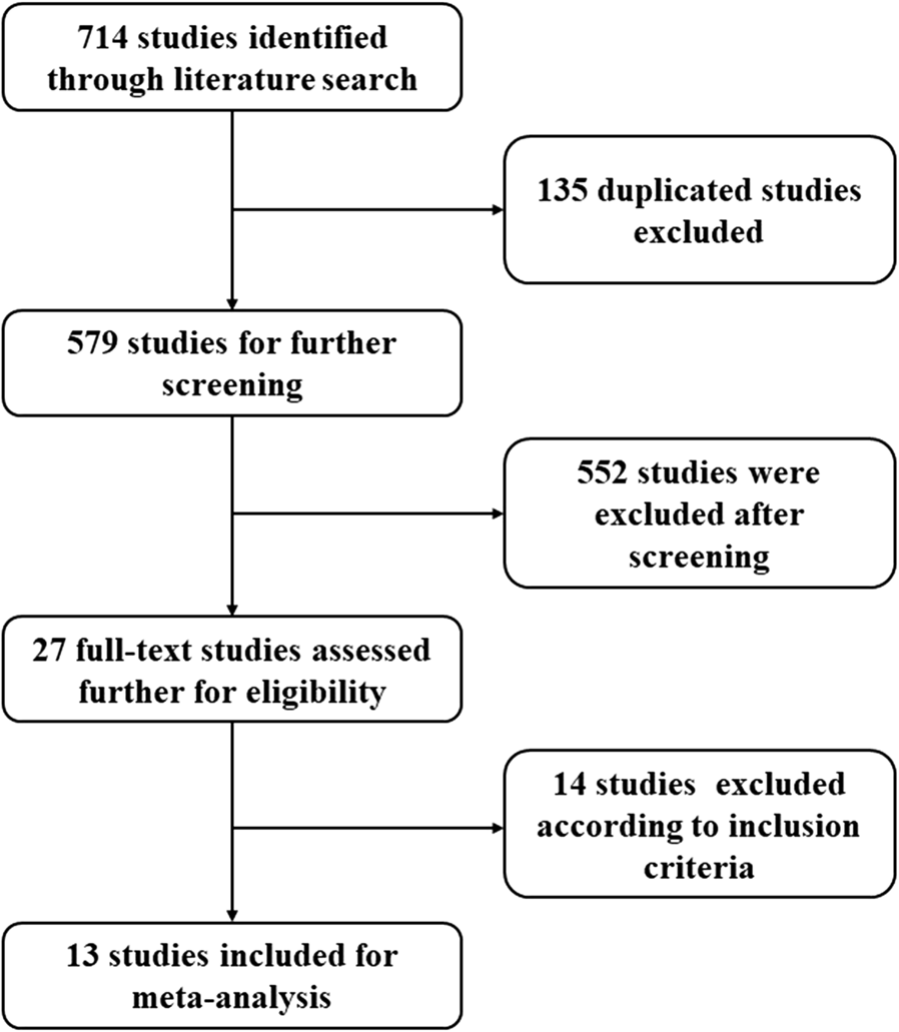
The flow diagraph of literature search and study selection

A total of 714 literatures were retrieved upon systematic literature search, among which 687 literatures were initially excluded through reviewing titles and abstracts. The remaining 27 studies were subsequently reviewed and screened according to our inclusion and exclusion criteria. Finally, 13 studies [13–17, 23–30] were included in our meta-analysis (Fig. 1). All the included studies were published between 2018 and 2020, of which all studies were retrospective design.

A total of 5279 patients which were from USA, Canada, Italy, France, Israel, and Japan were included in our meta-analysis. The diagnosed cancer types of these patients were mainly non-small cell lung cancer (NSCLC) (68.2%), melanoma (18.5%), and renal cell carcinoma (10.2%). With regard to BMI values, seven studies [13, 15–17, 24, 25, 28] stratified the BMI value into high BMI group and low BMI group by one cutoff value, four studies [14, 23, 27, 29] stratified the BMI values into normal BMI group (18.5 < BMI ≤ 24.9), overweight group (25.0 < BMI ≤ 29.9) and obese group (BMI ≥ 30) according to the WHO definition, one study [26] used both two methods to stratify BMI value. Notably, one study [30] stratifies BMI into 3 groups (BMI < 25, 25.0 ≤ BMI < 35, BMI ≥ 35), which was neither dichotomous nor stratifying BMI into normal overweight and obese groups. After carefully assessing this study, we found there was small proportion of patients in the group with BMI over 35. Based on this, we extracted the data comparing the two groups (BMI < 25, 25.0 ≤ BMI < 35). Ten studies reported the application of anti-PD-1/PD-L1 inhibitors, one study used the anti-CTLA4 inhibitors, and one study used the anti-PD-1/PD-L1 inhibitors or the anti-CTLA4 inhibitors. Main characteristics of 13 eligible studies including 15 cohorts are summarized in Table 1. Subsequently, NOS scale [18] was used to assess the quality of included studies. Among the 13 studies, seven studies had a score of 7, five studies had a score of 6, and one study had a score of 5. The results of quality assessment were displayed in Table 2.

**Table 1:** The baseline characteristics of included studies

Author	Year	Publication type	Source of analyzed data	Country	Cancer type	Sample size	Immunotherapy regimen	BMI stratification	Outcomes
Ichihara (cohort 1)	2020	Full text	Retrospective cohort	Japan	NSCLC	84	Cohort1: pembrolizumab	High: ≥ 22.0; low: < 22.0	OS, PFS
Ichihara (Cohort 2)	2020	Full text	Retrospective cohort	Japan	NSCLC	429	Cohort2: nivolumab, pembrolizumab, atezolizumab	High: ≥ 22.0; low: < 22.0	OS, PFS
Popinat	2019	Full text	Retrospective cohort	France	NSCLC	55	Nivolumab	High: ≥ 24.7; low: < 24.7	OS
Magri	2019	Full text	Retrospective cohort	Israel	NSCLC	46	Nivolumab	High: ≥ 24.6; low: < 24.6	OS
Kulkarni	2019	Abstract	Retrospective cohort	USA	NSCLC	148	Nivolumab	High: ≥ 25.0; low: < 25.0	OS, PFS
Kichenadasse	2019	Full text	NCT02031458 NCT01846416	USA, Australia	NSCLC	1434	Atezolizumab	Normal: 18.5–24.9 overweight: 25.0–29.9 obese: ≥ 30	OS, PFS, IRAEs
Zhi	2018	Abstract	Retrospective cohort	USA	NSCLC	703	Nivolumab, pembrolizumab	Normal: 18.5–24.9; overweight: 25.0–29.9; obese: ≥ 30	OS, PFS
Wang	2019	Full text	Retrospective cohort	USA	NSCLC Melanoma OC	250	PD-L1 inhibitor	High: ≥ 30; low: < 30	OS, PFS
Cortellini	2019	Full text	Retrospective cohort	Italy	NSCLC RCC melanoma	976	Pembrolizumab, nivolumab, atezolizumab	Definition1: high: ≥ 25.0; low: < 25.0 definition2: Normal: 18.5–24.9; overweight: 25·0–29.9; Obese: ≥ 30	OS, PFS, IRAEs
Naik	2019	Full text	Retrospective cohort	USA	Melanoma	139	PD-1 inhibitor	High: 25.0–35 low: < 25	OS, PFS
Richtig	2018	Full text	Retrospective cohort	Austria	Melanoma	76	Ipilimumab	High: ≥ 25.0; Low: < 25.0	OS, PFS
McQuade (cohort 1)	2018	Full text	NCT00324155& retrospective corhot	USA, Australia	Melanoma	207	Cohort 1: Ipilimumab plus dacarbazine	Normal: 18.5–24.9; overweight: 25.0–29.9; Obese: ≥ 30	OS, PFS, IRAEs
McQuade (Cohort 2)	2018	Full text	NCT00324155& Retrospective corhot	USA, Australia	Melanoma	329	Cohort 2: Pembrolizumab, nivolumab, atezolizumab	Normal: 18.5–24.9; Overweight: 25.0–29.9; Obese: ≥ 30	OS, PFS, IRAEs
Labadie	2019	Full text	Retrospective cohort	USA, Canada, Spain	RCC	90	Pembrolizumab, nivolumab, atezolizumab	Normal: 18.5–24.9 Overweight: 25.0–29.9 Obese: ≥ 30	OS, PFS
De Giorgi	2019	Full text	Retrospective cohort	Italy	RCC	313	Nivolumab	high: ≥ 25.0; low: < 25.0	OS

NSCLC, non-small cell lung cancer; RCC, renal cell cancer; OC, ovarian cancer; BMI, Body mass index; OS, overall survival; PFS, progression-free survival; IRAEs, immune-related adverse effects

**Table 2:** The quality assessment of included study using the Newcastle–Ottawa scale

Study	Selection				Comparability		Outcome			TOTAL	Quality
	REC	SNEC	AE	DO	SC	AF	AO	FU	AFU		
Ichihara 2020	1	1	1	1	0	0	1	0	1	6	Moderate
Wang 2019	1	1	1	1	0	0	1	0	1	6	Moderate
Popinat 2019	1	1	1	1	0	0	1	1	1	7	High
Naik 2019	1	1	1	1	0	0	1	1	1	7	High
Magri 2019	1	1	1	1	0	0	1	1	1	7	High
Labadie 2019	1	1	1	1	0	0	1	1	1	7	High
Kulkarni 2019	1	1	1	1	0	0	0	0	1	6	Moderate
Kichenadasse 2019	1	1	1	1	0	0	1	1	1	7	High
De Giorgi 2019	1	1	1	1	0	0	1	1	1	7	High
Cortellini 2019	1	1	1	1	0	0	1	0	1	6	Moderate
Richtig 2018	1	1	1	1	0	0	0	1	1	6	Moderate
Zhi 2018	1	1	1	1	0	0	0	0	1	5	Moderate
McQuade 2018	1	1	1	1	0	0	1	1	1	7	High

REC, representativeness of the exposed cohort; SNEC, selection of the non-exposed cohort; AE, ascertainment of exposure; DO, demonstration that outcome of interest was not present at start of study; SC, study controls for age, sex; AF, study controls for any additional factors (chemoradiotherapy, curative resection); AO, assessment of outcome; FU, follow-up long enough (36 M) for outcomes to occur; AFU, adequacy of follow-up of cohorts. “1” means that the study is satisfied the item and “0” means the opposite situation

### BMI and overall survival (OS)

**Fig. 2:**
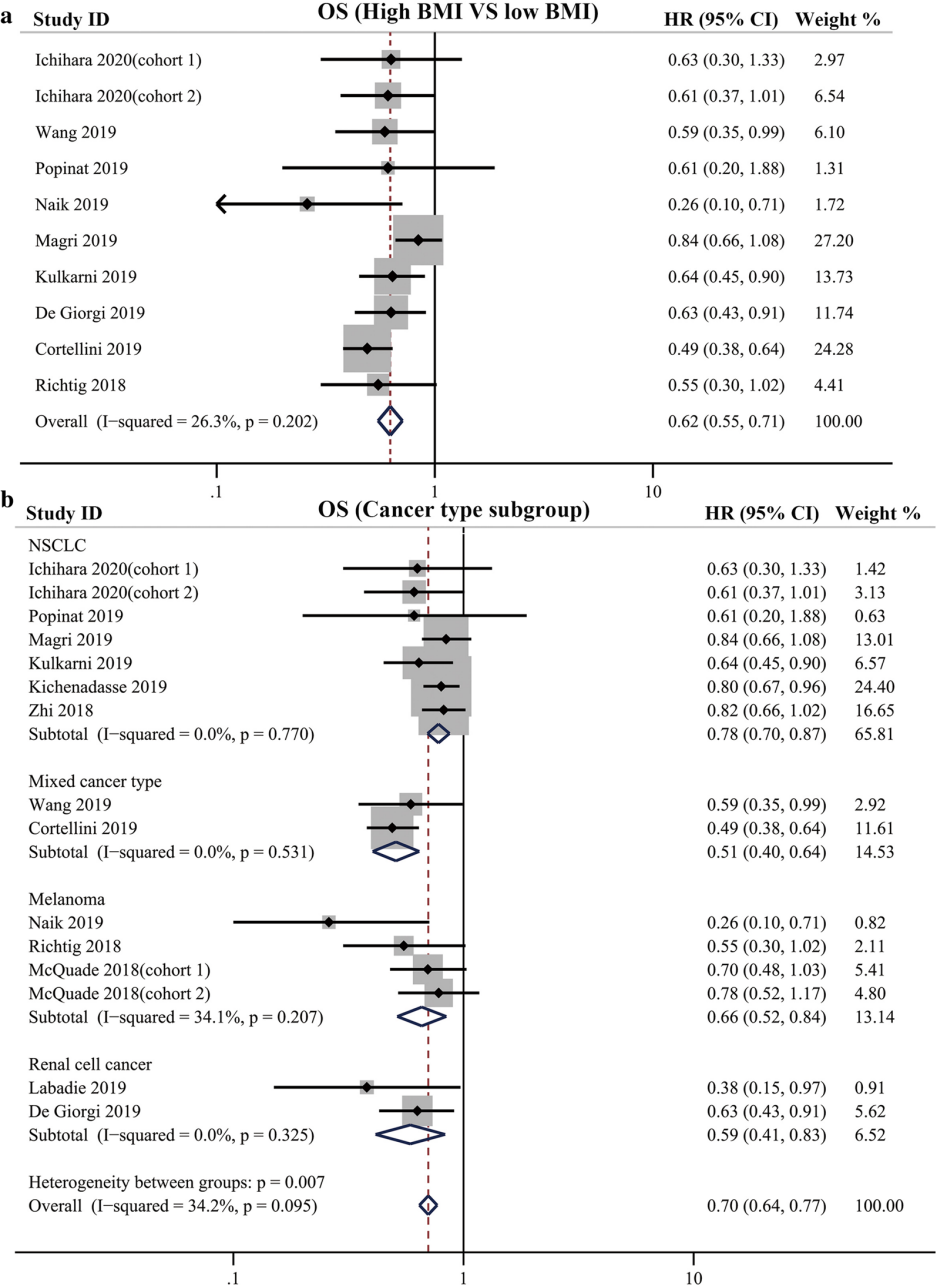
**a** Pooled analysis showing associations between BMI and OS in cancer patients receiving immune checkpoint inhibitors treatment; **b** Subgroup analysis of associations between BMI and OS based on cancer type

Firstly, we analyzed the pooled HR for OS in 9 studies which stratified the BMI value into high BMI group and low BMI group. There were 10 cohorts reporting the HR for OS in these 9 studies in the setting comparing survival difference between patients high BMI and those with low BMI. The pooled analysis showed there is an improved OS in patients with high BMI compared with patients with low BMI after receiving ICIs therapy (HR = 0.62, 95% CI 0.55–0.71, *P* < 0.0001) (Fig. 2a). There was no significant heterogeneity across the 10 cohorts in terms of OS (I^2^ = 26.3%, *P* = 0.202). The funnel plot showed no obvious publication bias regarding OS among these studies (z = 1.25, Begg’ s test *P *= 0.21) (Additional file 1).

Subsequently, we included the studies that dichotomized BMI into high and low group and those studies with obese versus normal comparisons, and performed subgroup analysis based on cancer type. The results showed that patients with high BMI possessed improved OS (NSCLC subgroup: HR = 0.78, 95% CI 0.70–0.87, *P *< 0 0.001, I^2^ = 0%; Mixed cancer type subgroup: HR = 0.51, 95% CI 0.40–0.64, *P *< 0 0.001, I^2^ = 0%; Melanoma subgroup: HR = 0.66, 95% CI 0.52–0.84, *P *= 0 0.001, I^2^ = 34.1%; Renal cancer subgroup: HR = 0.59, 95% CI 0.41–0.83, *P *= 0 0.003, I^2^ = 0%) (Fig. 2b).

**Fig. 3:**
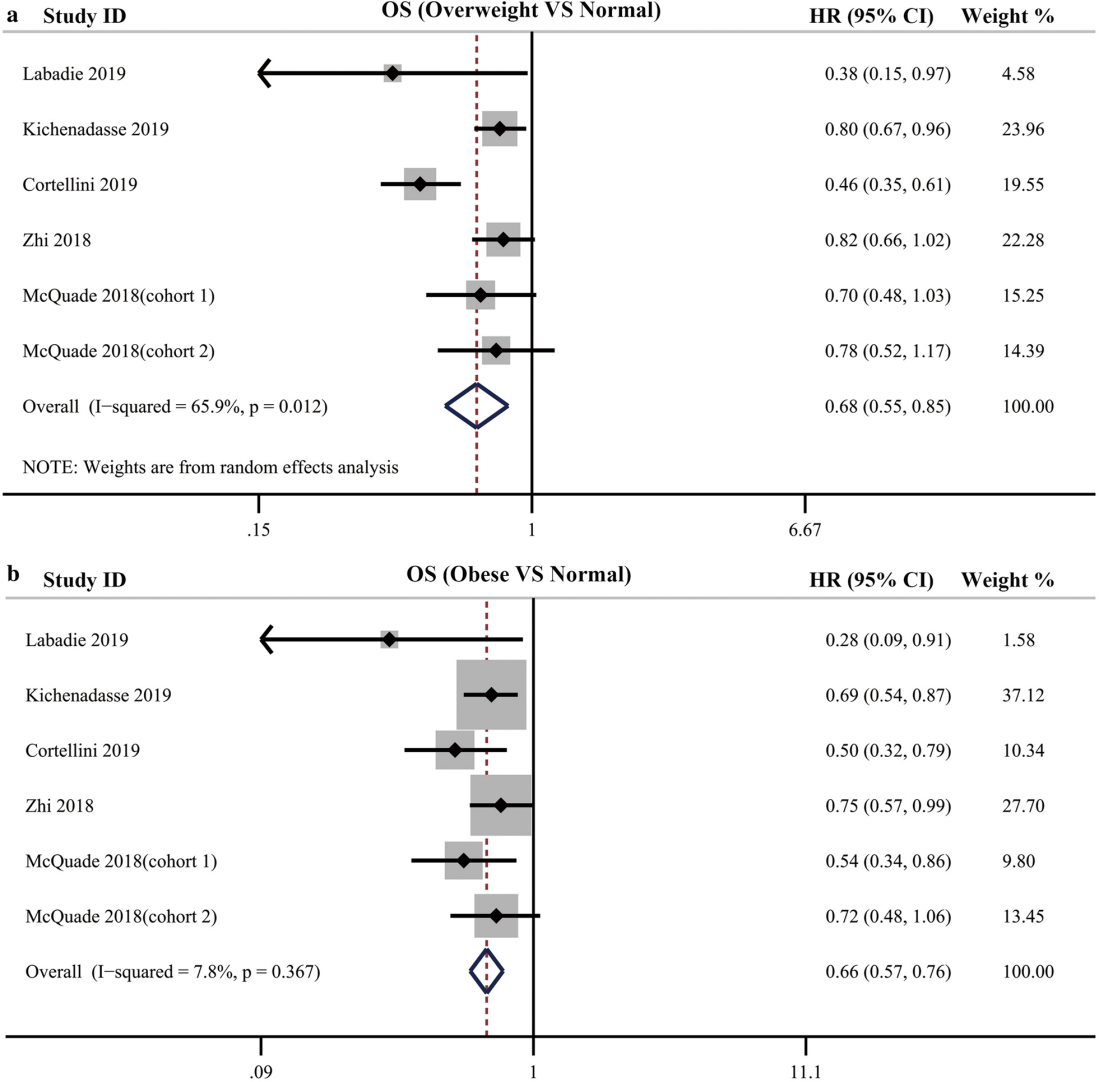
**a** Pooled analysis showing improved OS in overweight cancer patients compared with normal patients receiving immune checkpoint inhibitors treatment; **b** Pooled analysis showing improved OS in obese cancer patients compared with normal patients receiving immune checkpoint inhibitors treatment

Some included studies stratified the BMI values into normal BMI group, overweight group and obese group according to the WHO definition. In these studies, the OS for patients with overweight BMI, obese BMI was respectively compared with normal BMI. The pooled analysis showed that OS was significantly different between the patients with normal weight, overweight, and obesity treated with ICIs, with improved OS for patients with overweight BMI values (HR = 0.68; 95% CI, 0.55–0.85; *P *= 0.001; I^2^ = 65.9%) with significant heterogeneity and obesity BMI values (HR = 0.66; 95% CI, 0.57–0.76; *P *< 0.001; 7.8%) compared with patients with normal BMI without significant heterogeneity (Fig. 3).

### BMI and progression-free survival (PFS)

**Fig. 4:**
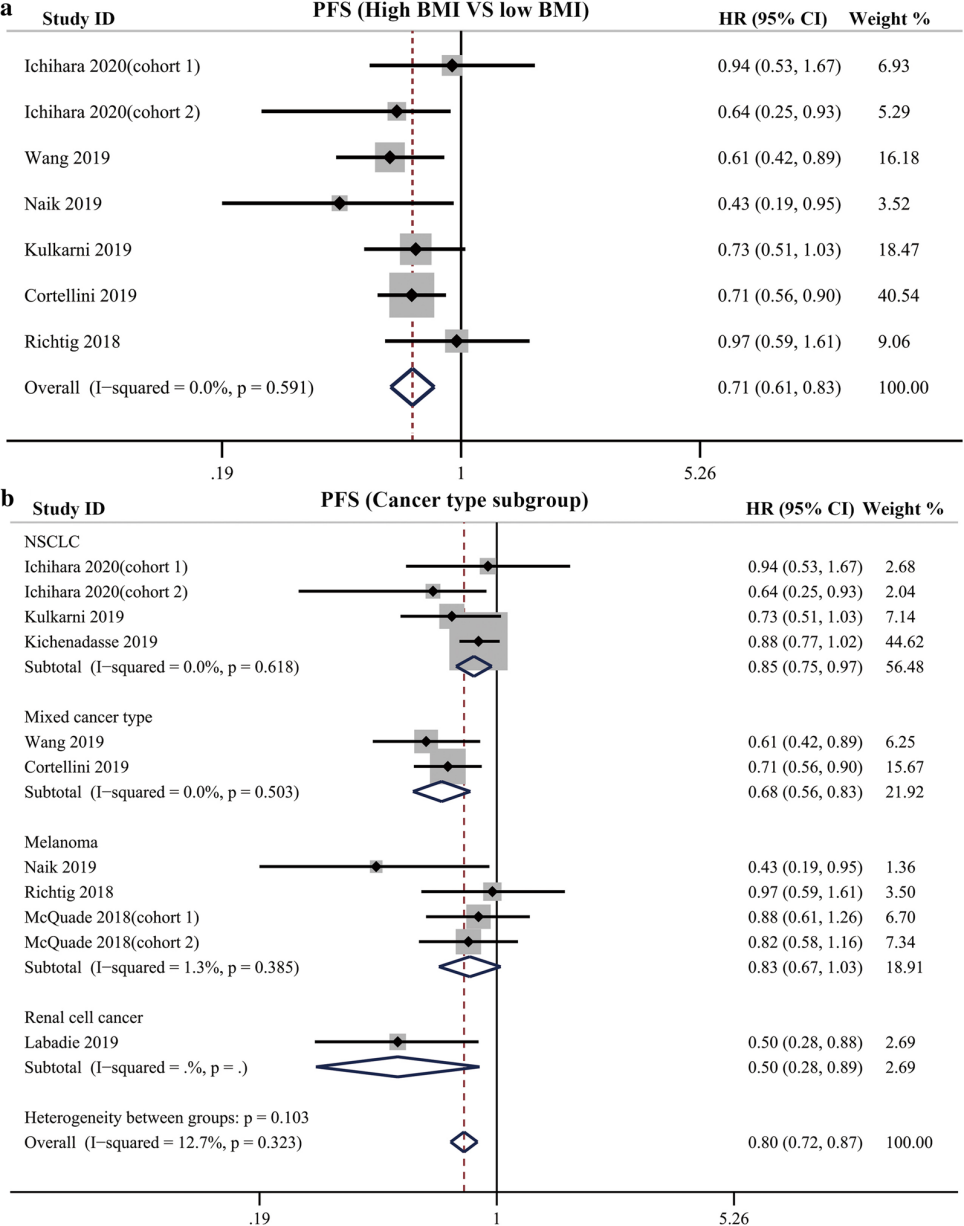
**a** Pooled analysis showing associations between BMI and PFS in cancer patients receiving immune checkpoint inhibitors treatment; **b** Subgroup analysis of associations between BMI and PFS based on cancer type

Among the 9 studies which stratified the BMI value into high BMI group and low BMI group, 6 studies including 7 cohorts reported the HR for PFS. The pooled analysis indicated that high BMI was associated with improved PFS in cancer patients receiving ICIs therapy (HR = 0.71, 95% CI 0.61–0.83, *P *< 0.0001), with no significant heterogeneity among these studies (I^2^ = 0%, *P* = 0.591) (Fig. 4a). The results of Begg’s and Egger’s tests showed that no significant publication bias was found in the overall analysis of PFS (*P*_Begg’s_ = 0.548, *P*_egger’s_ = 0.878; Additional file 1).

We perform subgroup analyses among the studies that dichotomized BMI into high and low group and those studies with obese versus normal comparisons based on cancer type, showing improved PFS was found in patients with high BMI compared with those with low BMI (NSCLC subgroup: HR = 0.85, 95% CI 0.75–0.97, *P *= 0 0.012, I^2^ = 0%; Mixed cancer type subgroup: HR = 0.68, 95% CI 0.56–0.83, *P *< 0 0.001, I^2^ = 0%; Melanoma subgroup: HR = 0.83, 95% CI 0.67–1.03, *P *= 0 0.087, I^2^ = 1.3%; Renal cancer subgroup: HR = 0.50, 95% CI 0.28–0.89, *P *= 0 0.018, I^2^ = 0%) (Fig. 4b).

**Fig. 5:**
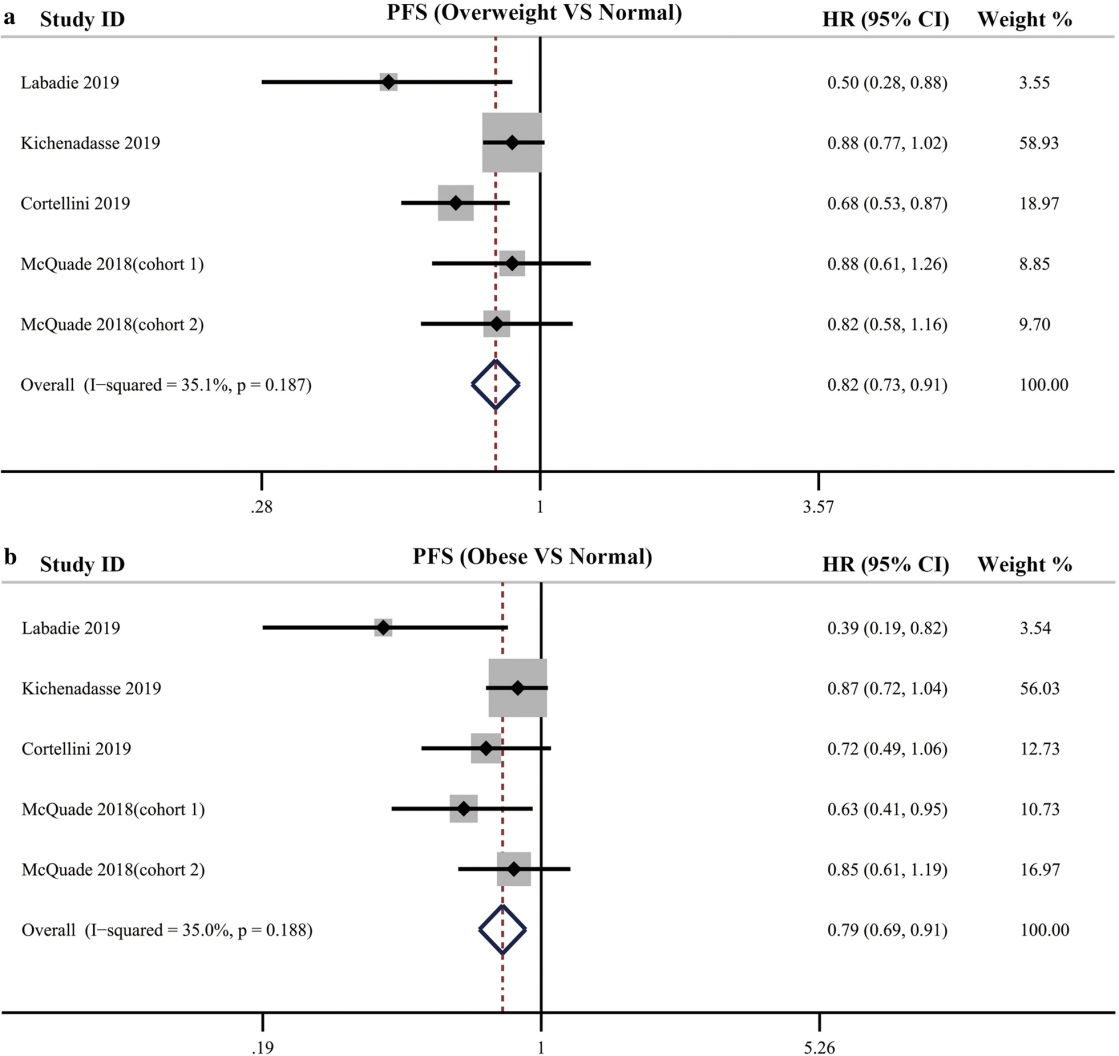
**a** Pooled analysis showing improved PFS in overweight cancer patients compared with normal patients receiving immune checkpoint inhibitors treatment; **b** Pooled analysis showing improved PFS in obese cancer patients compared with normal patients receiving immune checkpoint inhibitors treatment

Pooled analysis for PFS was also performed in the setting of comparing obese, overweight patients with normal patients. The results showed, compared with normal patients, overweight and obese patients showed an improved PFS (overweight vs normal: HR = 0.82, 95% CI = 0.73–0.91, *P *< 0 0.001, I^2^ = 35.1%; obese vs normal: HR = 0.79, 95% CI = 0.69–0.91, *P *= 0 0.001, I^2^ = 35.0%) (Fig. 5).

### BMI and immune-related adverse effects (IRAEs)

**Fig. 6:**
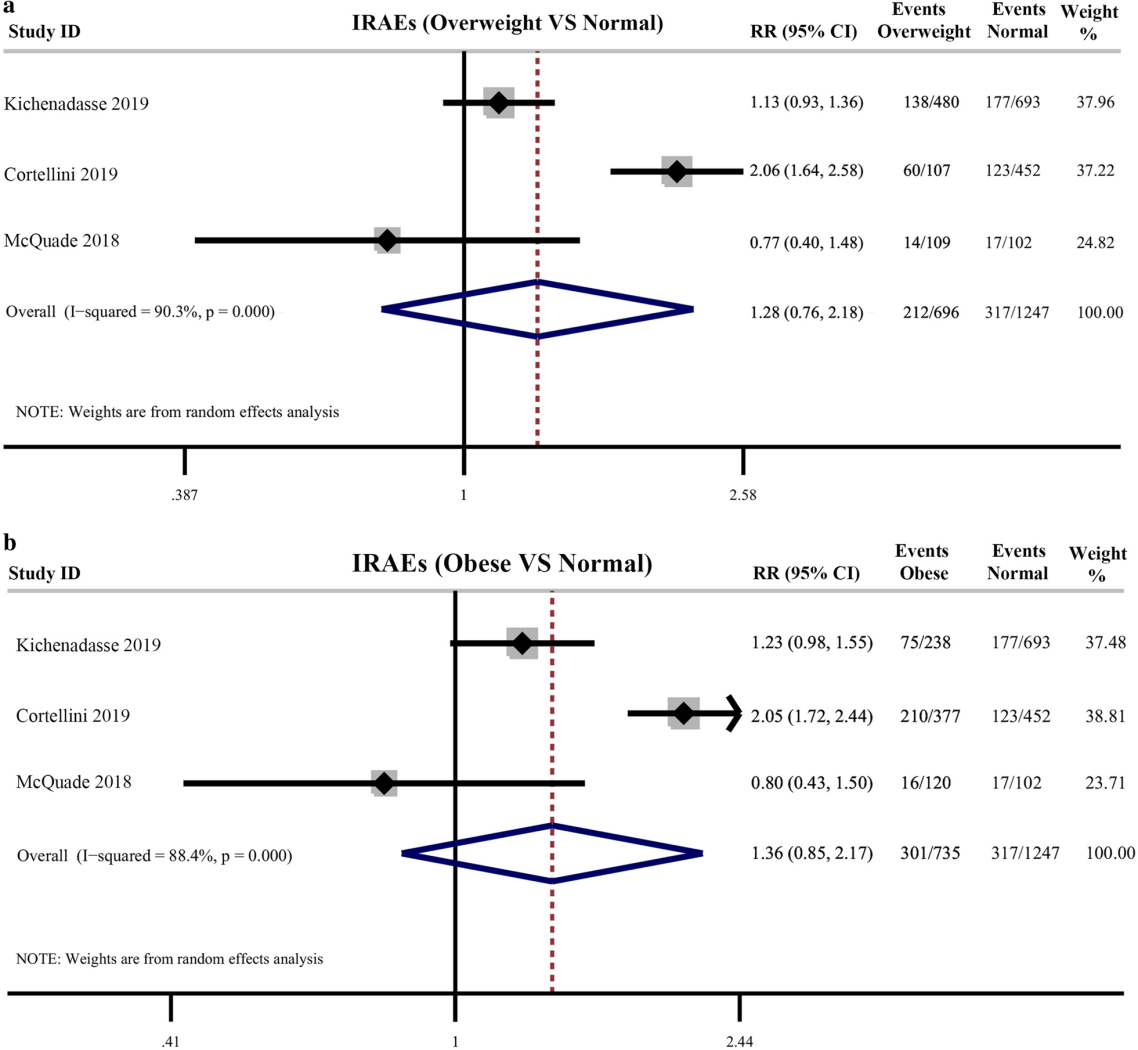
**a** Pooled analysis showing incidence difference of immune-related adverse effects between overweight cancer patients and normal patients receiving immune checkpoint inhibitors treatment; **b** Pooled analysis showing incidence difference of immune-related adverse effects between obese cancer patients and normal patients receiving immune checkpoint inhibitors treatment

There were 3 studies reporting the incidence of all grades IRAEs. The pooled results showed that there was no significant difference between the incidence of all grade IRAEs between obese, overweight patients and normal patients (Overweight vs Normal: pooled RR = 1.28, 95% CI 0.76– 2.18, *P *= 0.356; I^2^ = 90.3%; Obese vs Normal: pooled RR = 1.36, 95% CI 0.85–2.17, *P *= 0.207; I^2^ = 88.4%) with significant heterogeneity between studies (Fig. 6).

## Discussion

The advent of immune-checkpoint inhibitors (ICIs) treatment has changed the therapeutic paradigm for cancer and brought survival benefit for some patients [2]. However, there were considerable amounts of patients not responding to these treatments, and few factors exists to identify those patients who will benefit from ICIs treatment among cancer patients. Recently, several patient-associated demographic characteristics are being evaluated to estimate the efficacy of ICIs [10, 31–34]. Among these characteristics, patients’ gender and BMI values were investigated by some researchers. A recent meta-analysis evaluated the association between patients’ sex and ICIs efficacy, revealing ICIs can improve survival for patients of both sexes, but men have a larger treatment effect than women [10]. In our current meta-analysis, we evaluated the association between BMI and survival of cancer patients receiving ICIs treatment, and our findings suggest that high BMI have a positive association with improved OS and PFS for cancer patients.

To our knowledge, this is the first meta-analysis to clearly evaluate the association between patients’ BMI values and the efficacy of ICIs for cancer patients. Through a systemic literature search and screening, our current meta-analysis included 12 eligible studies containing 5140 patients to assess the impact of BMI on the efficacy of ICIs after receiving ICIs treatment. Our results revealed that higher BMI is associated with improved OS and PFS in the setting of comparing high BMI with low BMI for cancer patients treated with ICIs, and both obese and overweight patients possessed survival benefit in OS and PFS in the setting of comparing obese and overweight BMI with normal BMI. Similarly, subgroup analysis based on cancer type also showed improved OS and PFS in patients with NSCLC and mixed cancer type except for the PFS in melanoma subgroup. The discrepancy in melanoma may be explained by few studies included in this subgroup. In the future, more studies were required to clearly investigate the association between BMI and ICIs efficacy in patients with melanoma after ICIs treatment.

Higher BMI has correlated with a survival advantage to ICIs therapy for cancer patients as revealed by our meta-analysis. A recent study suggested that obesity is associated with improved PFS and OS compared with those outcomes in melanoma patients with normal BMI after receiving ICIs treatment [29]. Another study performed in NSCLC patients also suggested high BMI was associated with improved PFS and OS in patients treated with ICIs [14]. Currently, the mechanisms through which BMI impact survival outcomes after ICIs treatment is just beginning to be understood. The possible explanation may be that obesity may promote the formation of a systemic meta-inflammation and result in a dysregulated immune response. A recent study [35] pointed that adipocytes in adipose tissues could release several proinflammatory cytokines and chemokines to form and maintain inflammatory environment, which may potentiate the effect of immune checkpoint inhibitors. In addition, basic experimental study also found obesity could induce T cell dysfunction and increase the number of PD-1 positive T cells in the peripheral blood and tumors, which may partially explain the influence of BMI on the efficacy of ICIs [15].

As revealed in our study and others, BMI values is positively associated with improved survival outcomes for cancer patients receiving ICIs therapy. As BMI is calculated through the whole-body weight, there is still some limitation by using BMI as a surrogate of adiposity. BMI may not be sufficient enough to reflect the complex body composition. Some other measures such as cross-sectional imaging or Dual-energy X-ray absorptiometry (DEXA) showed advantages on directly discriminating and measuring the body compositions like fat tissue, muscle tissue, bone mineral density. A recent meta-analysis [36] evaluated the association between computerized tomography (CT) based quantification of subcutaneous, visceral, skeletal muscle tissues and cancer patient outcomes, demonstrating that high visceral adipose tissue and reduced skeletal muscle correlated with poorer survival. Chambard et al. [37] reported sarcopenia measured via DEXA was associated with poorer survival in lung cancer patients with bone metastasis. Further studies are required the evaluated the detailed body composition changes during receiving ICIs treatment and the association between body composition changes and survival outcomes among cancer patients with ICIs treatment.

We observed that the association between high BMI and improved survival in cancer patients with ICIs therapy. Our results may help encourage the emergence of prospective interventional trials which randomly stratify the patients into groups according to BMI value and treat patients with ICIs. Through such prospective interventional trials, researchers could better understand the influence of BMI on ICIs efficacy. Meanwhile, when carrying out randomized control trials to evaluate the efficacy of ICIs in cancer patients, investigators can also stratify the invention and control group into subgroups by BMI values and comparing the efficacy difference based on subgroup analysis. Apart from the influence on ICIs efficacy, higher BMI also showed association within other treatment types. For cancer patients receiving operation therapy, studies reported that high BMI was positively associated with a higher risk of postoperative complications, but it does not influence the prognosis of cancer patients [38, 39]. One of our included studies also investigated the relationship between BMI and outcomes for patients treated with docetaxel, and they found no significant association between BMI and survival outcome [14]. In the future, more studies are needed to explore the effect of BMI on chemotherapy efficacy for cancer patients.

The association between BMI and IRAEs in patients receiving ICIs treatment has been variably reported [14, 26, 29]. In our current study, the pooled analysis showed that patients with obesity had no difference in the incidence of any grade of IRAEs compared with normal patients. In our current meta-analysis, only 3 of our included studies reported the association between BMI and incidence of IRAEs. Future research using large datasets from ICIs-related trials were required to evaluate the association between obesity and incidence of IRAEs.

In our included studies, one study reported the association between BMI and the ICIs efficacy respectively in male and female patients. The findings in this study suggested that the relationship between BMI and outcomes in cancer patients might vary by gender, with a survival advantage observed in obese men treated with ICIs, but not in obese women. This difference can be partially explained by different response of male and female patients receiving ICIs. A recent meta-analysis [10] showed male patients treated with ICIs had a significantly reduced risk of death compared with men treated in control groups and the benefit obtained with ICIs treatment compared with control groups was smaller in female patients, suggesting men showed a significant advantage from ICIs treatment compared with female patients. However, another gender-based analysis demonstrated that overweight female patients possessed obvious clinical benefit from immunotherapy compared with the male patients [26]. This inconsistent conclusion suggested that future large-scale studies are required to evaluate association between ICIs efficacy and BMI in patients with different sex.

Although we found relationship between high BMI values and improved prognosis for cancer patients with ICIs therapy, absence of a homogeneous cut off value stratifying the BMI values might have affected the results. In the set of a single cut-off value, it might not be enough to avoid the prognostic negative role of cancer cachexia which may occur among patients with low BMI even under normal level. Meanwhile, some studies evaluated the survival difference between Obese patients (BMI greater than 30) and normal-weight ones, while some studies evaluated survival outcomes between overweight (BMI greater than the cutoff value) and non-overweight patients (BMI under the cutoff value). These non-overweight patients may also include underweight patients whose BMI value was under 18.5. This also may had influenced the results. In the future, studies focusing on BMI and ICIs efficacy could set more cutoff values and more comparisons to eliminate the influence some profound factors like cancer cachexia etc.

Some limitations still existed in current study: (1) The results of this meta-analysis were based on data from published studies and the detailed individual data was not obtained for analysis. (2) Although the association between BMI and IRAEs was assessed in our analysis, the included studies were fewer and significant heterogeneity was observed among these studies. Future studies were required to clearly investigate the relationship between BMI and the incidence of IRAEs. (3) our study could not evaluate the association between BMI and tumor response to ICIs because of fewer included data. Therefore, future studies are needed to assess this potential association. (4) Some studies included in this meta-analysis used BMI cutoff value to stratify high BMI and low BMI groups. However, some difference between the BMI cutoff value may increase the heterogeneity between studies. In the future, a more standard cutoff value definition is required to stratify BMI and reduce the heterogeneity between studies. (5) Different ICI classes was used in the included studies. For example, some studies used the anti-CLTA4 immunotherapy, some used anti PD1/PD-L1 drugs, and part of studies used both kinds. These differences may increase the heterogeneity between studies.

## Conclusions

In summary, current meta-analysis showed that there is significant association between BMI and survival outcomes for patients receiving ICIs treatment. An improved OS and PFS were observed in patients with high BMI after receiving ICIs treatment compared with patients with low BMI, also in obese and overweight patients compared with normal patients after ICIs therapy. However, no significant association between BMI and incidence of IRAEs was found in cancer patients after ICIs treatment.

## Supplementary information

**Additional file 1:** Funnel plot of overall survival; B. Funnel plot of progression-free survival.

## Data Availability

All data generated or analysed during this study are included in this published article and its Additional file 1.

## Abbreviations

ICIs: Immune checkpoint inhibitors
PD-1: Programmed cell death 1
PD-L1: Programmed cell death 1 ligand 1
CTLA-4: Cytotoxic T lymphocyte-associated antigen 4
TILs: Tumor infiltrating lymphocytes
BMI: Body mass index
OS: Overall survival
PFS: Progression-free survival
NOS: Newcastle–Ottawa scale
HR: Hazard ratio
RR: Relative risk
IRAEs: Immune-related adverse effects
