# Supplementary figures and images for: Association between body mass index and survival outcomes for cancer patients treated with immune checkpoint inhibitors: a systematic review and meta-analysis
Source: J Transl Med. 2020 Jun 12;18:235. doi: 10.1186/s12967-020-02404-x (PMC7291531; doi:10.1186/s12967-020-02404-x)

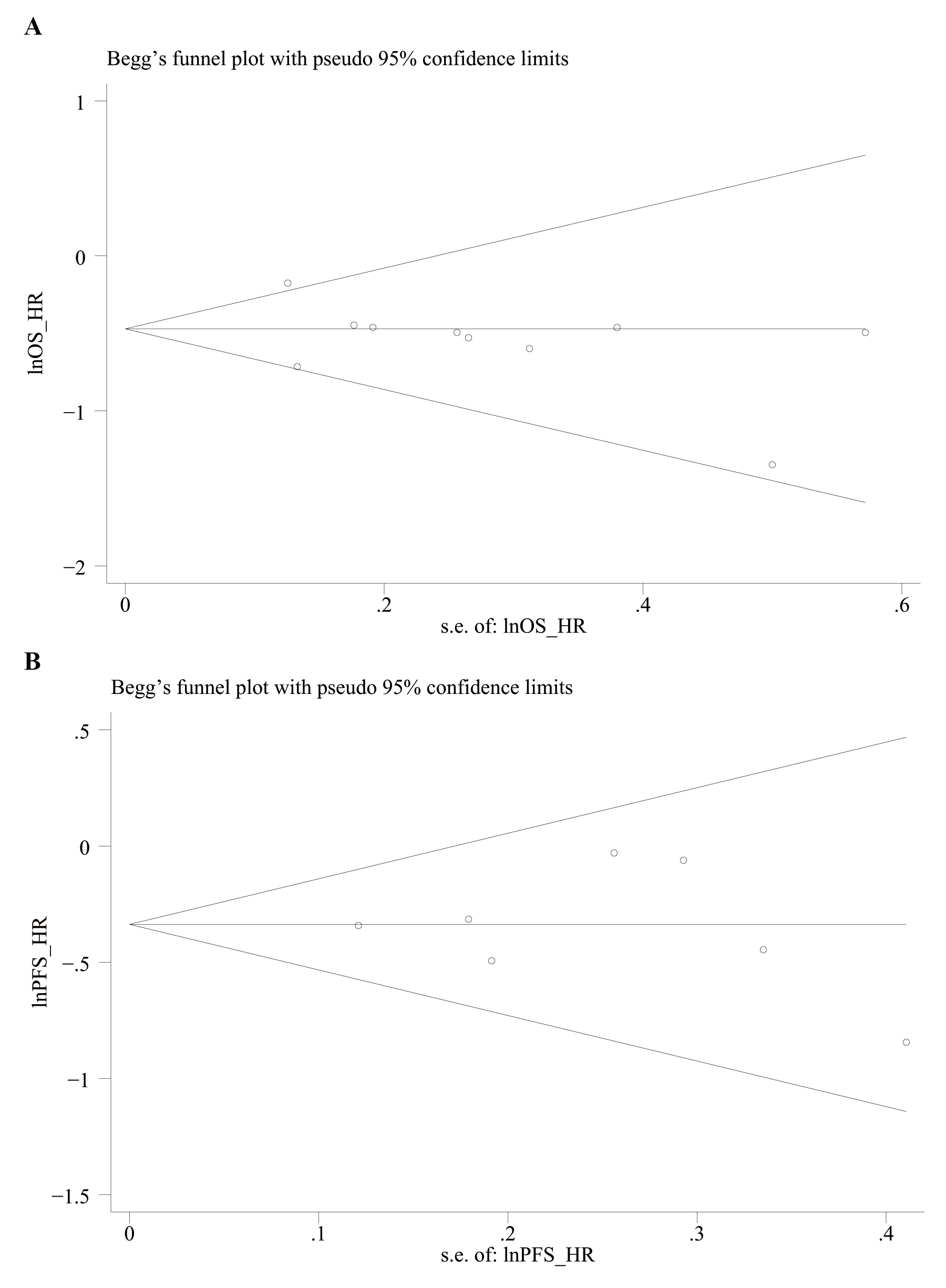

Supplement: Supplementary file 1 — Additional file 1: Funnel plot of overall survival; B. Funnel plot of progression-free survival. [file 12967_2020_2404_MOESM1_ESM.jpg]
